# Early Changes in Quality of Life and Symptom Burden Following Cancer‐Directed Treatment Among Head and Neck Cancer Patients in Bangladesh: Findings From a Prospective Pilot Study

**DOI:** 10.1002/hsr2.73228

**Published:** 2026-09-13

**Authors:** Md. Jahirul Islam, A.B.M. Alauddin Chowdhury, Md. Imdadul Haque, Md. Khalid Mahmud, Kawsar Ahmed, Hafiz T. A. Khan, Salim Khan

**Affiliations:** ^1^ Department of Public Health, Faculty of Health and Life Sciences Daffodil International University, Daffodil Smart City Dhaka Bangladesh; ^2^ Department of Public Health City South Campus, Birmingham City University Birmingham UK; ^3^ Department of Family Medicine and Public Health Spencer Fox Eccles School of Medicine, University of Utah Salt Lake City Utah USA; ^4^ National Institute of Cancer Research & Hospital, Mohakhali Dhaka Bangladesh; ^5^ Central South University Changsha China; ^6^ Public Health Group University of West London United Kingdom; ^7^ Oxford Institute of Population Ageing University of Oxford Oxford UK

**Keywords:** Bangladesh, cancer‐directed treatment, early change, head and neck cancer, quality of life

## Abstract

**Background and Aims:**

Evidence on early quality of life (QoL) outcomes among patients with head and neck cancer (HNC) remains limited in low‐resource settings like Bangladesh. This prospective pilot study assessed changes in QoL, functioning, and symptom burden following initiation of cancer‐directed treatment (CDT) among patients with HNC in Bangladesh.

**Methods:**

A prospective observational pilot study was conducted among 102 consecutively recruited patients with HNC from three public cancer care hospitals located in Dhaka City, Bangladesh. Ninety‐one patients were reached at follow‐up, of whom 57 completed QoL assessment following a mean of 7.4 ± 2.2 weeks of treatment initiation. QoL was assessed using the European Organization for Research and Treatment of Cancer Quality of Life Questionnaire Core 30 (EORTC‐QLQ‐C30). Linear mixed‐model analyses were employed to evaluate longitudinal changes and identify factors associated with QoL outcomes.

**Results:**

Of 91 followed‐up patients, 16.5% of patients died, and 27.5% did not start CDT, including 25% of survivors. Global quality of life (GQL) among 57 finally assessed patients remained impaired and unchanged over time (β = −0.15, *p* = 0.95). Overall functioning declined markedly (β = −5.87, *p* = 0.03), primarily mediated by deteriorated physical and role functions, along with significantly increased fatigue, nausea/vomiting, and insomnia. Younger than 50 years older and better social functioning were positively associated with QoL, whereas clinical conditions such as laryngeal or pharyngeal cancers, fatigue, pain, and financial difficulties were independently associated with poorer QoL.

**Conclusion:**

Patients with HNC in Bangladesh experienced an extensive burden of deteriorated QoL, functioning, and increased symptom severity during the early treatment period, besides considerable treatment gaps and mortality. Our findings underscore the necessity of addressing treatment gaps, symptom management, and supportive care. More studies are recommended to support policy implications and implementation regarding early identification, diagnosis, and treatment of HNC in resource‐limited settings.

## 1 Introduction

Head and neck cancer (HNC) remains one of the public health concerns, with an estimated 0.95 million new cases and 0.48 million deaths reported worldwide in 2022 [1, 2, 3]. Low‐ and middle‐income countries (LMICs), including countries of Southeast Asia, contribute disproportionately higher burden of HNC to the global estimates. This is mainly attributed to widespread consumption of tobacco, areca nut, and alcohol [2, 4, 5, 6]. In Bangladesh, HNC accounts for nearly 17% among all cancers, with around 37,000 annual incidences [1, 7, 8]. Despite this substantial burden, Bangladesh continues to face major challenges in cancer prevention, early diagnosis, treatment accessibility, and supportive care delivery [7, 8, 9, 10, 11, 12].

Quality of life (QoL), also referred to as health‐related quality of life (HR‐QoL), is a subjective measure used by healthcare providers to assess a multidimensional construct that reflects patients' physical, psychological, social, and functional well‐being in relation to disease and treatment [13, 14, 15]. Among all cancer groups, patients with HNC are considered particularly vulnerable to poor QoL [2, 6] because the disease [6, 16, 17] and its treatment [16, 18, 19, 20, 21, 22] directly affect essential functions such as speaking, swallowing, breathing, chewing, and eating [16, 18, 19, 21, 23, 24, 25, 26, 27, 28, 29]. Previous studies have consistently demonstrated substantial deterioration in physical functioning, psychological states, symptom burden, and financial hardship among patients with HNC [16, 18, 23, 24, 25, 26, 30, 31, 32, 33, 34, 35].

Both socio‐demographic [27, 33, 36, 37] and clinical factors [6, 16, 17] influence QoL outcomes in HNC. Older age [18, 28, 29, 30], gender [21, 27, 31, 38], educational attainment [30], financial difficulties [4, 18, 25, 26, 29, 33, 34, 35, 39], and tobacco use [21, 24, 31] affect pre‐ and post‐treatment functioning [15, 16, 18, 19, 23, 24, 25, 29] and QoL [15, 16, 17, 18, 22, 23, 24, 28, 37] among patients with HNC. In addition, mixed treatment modalities like surgery combined with chemoradiotherapy (CRT) or radiation and surgery are frequently correlated with severe symptoms such as pain [15, 34, 39, 40], nausea/vomiting [15, 16, 18, 41], cancer‐related fatigue [42], and insomnia [15, 16, 18, 41], all of which contribute to deteriorated QoL. Further, impaired HR‐QoL negatively affects survivorship among patients with HNC [17, 43, 44, 45].

Bangladeshi patients with cancer commonly confront delayed identification of disease, treatment disruption, and financial adversity primarily due to resource‐limited healthcare settings, weak awareness, lower levels of education, and geographical remoteness in accessing specialized care [8, 9, 11, 46, 47]. In addition, there is scarce evidence on patient outcomes among Bangladeshi patients with HNC. Previous studies among Bangladeshi cancer patients have mostly concentrated on mixed cancer samples and reported poorer QoL across physical, social, emotional, and spiritual domains, with financial sufferings and progressive disease stages identified as major factors [48, 49, 50, 51]. A locally published prospective study among patients with advanced‐stage HNC also revealed limited improvement in functional performance using the European Cooperative Oncology Group (ECOG) performance scale, with ECOG‐1 increasing from 21.9% to 26.6% and ECOG‐2 decreasing from 65.6% to 60.9% following treatment [10]. Nevertheless, longitudinal evidence specifically evaluating QoL changes and associated factors among patients with HNC in Bangladesh is lacking.

Therefore, this prospective observational pilot study aimed to assess early changes in QoL, functioning, and symptom burden following cancer‐directed treatment among patients with HNC in Bangladesh and to identify sociodemographic and clinical factors associated with QoL outcomes.

## 2 Materials and Methods

### 2.1 Study Design and Settings

This prospective observational pilot study was conducted to explore early clinical outcomes among Bangladeshi patients with HNC, including functioning, symptom burden, and QoL within 3 months following diagnosis. Patients with HNC who attended three public tertiary cancer care hospitals, namely the National Institute of Cancer Research and Hospital (NICRH), the National Institute of Ear, Nose, and Throat (NIENT), and Dhaka Dental College and Hospital (DDCH) in Dhaka, Bangladesh, were included in this study. Participants were prospectively followed from baseline assessment at diagnosis to follow‐up assessment after initiation of CDT.

### 2.2 Participation Criteria

Newly diagnosed patients with HNC aged 18 years and older were eligible for possible enrolment. Patients who were severely ill, unable to communicate, unwilling to participate, or had a previous history of receiving cancer treatment were excluded from the study. Histopathologically confirmed [52] patients with HNC who attended inpatient and outpatient units dedicated to HNC within oncology departments of participating hospitals during the study period were consecutively recruited.

### 2.3 Participants Recruitment and Follow‐up

A total of 102 patients with HNC were consecutively enrolled at baseline across the participating hospitals (75 from NICRH, 20 from NIENT, and 7 from DDCH). The assessment was conducted on two separate occasions: right after diagnosis (baseline) and a mean duration of 7.4 ± 2.2 weeks following treatment initiation (follow‐up). Follow‐up information was obtained from 91 participants and/or their caregivers, and 57 who initiated CDT completed follow‐up assessment and were included in the longitudinal QoL analyses. Among baseline patients, 45 had been lost to follow‐up due to not initiating CDT (19 cases), being unresponsive to follow‐up communications (11 cases), or death (15 cases).

### 2.4 Data Collection Procedures

Data were collected using a semi‐structured interviewer‐administered questionnaire and review of relevant medical records. Baseline information included sociodemographic factors such as age, gender, marital status, educational attainment, employment, residential location, socioeconomic status, and tobacco exposure. Clinical characteristics included body mass index (BMI), disease location, disease stage with tumor, node, and metastasis (TNM), and treatment modality. TNM staging was based on the 8th edition of the American Joint Committee on Cancer (AJCC) [52].

The QoL data were collected using the Bengali EORTC‐QLQ‐C30 questionnaire that the EORTC provided at our request (https://qol.eortc.org/questionnaires/). The Bengali version of the EORTC‐QLQ‐C30 questionnaire was also used in evaluating QoL of cancer patients in Bangladesh [48].

Baseline data were collected by administering a Bengali‐scripted questionnaire face‐to‐face. Participants were followed up through telephone communication to obtain information on survival status, treatment initiation, treatment modalities, and QoL outcomes. Baseline data were collected in July‐August 2021, while follow‐up was conducted in November 2021.

### 2.5 Measurement of Quality of Life

Quality of life was measured using EORTC QLQ‐C30, a validated cancer‐specific tool comprising GQL, functioning, and symptom domains. The QLQ‐C30 consists of two GQL items, 15 functional items grouped into five scales (physical, role, emotional, cognitive, social), and 13 symptom items divided into three multi‐item scales (fatigue, nausea and vomiting, pain) and six single scales (dyspnea, appetite loss, constipation, diarrhea, insomnia, financial difficulties). All these items pertain to the respondent's condition in the past 7 days. Items 1–28 are scored on 4‐point Likert‐type categorical scales (1 = not at all, 4 = very much), and items 29–30 are scored on 7‐point (1 = very poor, 7 = excellent) numeric scales.

All raw scores are linearly transformed into a 0–100 scale. Higher mean scores on GQL and functioning scales indicate higher QoL and functioning, whereas higher symptom scores reflect greater symptom burden [18, 19, 39, 40, 42]. For this study, overall functioning scores were calculated by averaging five functioning domains, while general cancer‐related symptom (GC‐RS) scores were obtained by averaging nine symptom scales [53, 54].

### 2.6 Data Analysis

Data was analyzed using the Statistical Package for the Social Sciences (SPSS) version 26.0 (Armonk, NY: IBM Corp., USA). Descriptive statistics were presented as frequencies, percentages, means with standard deviation (SD), and median with interquartile ranges (Q1, Q3). Linear mixed‐model (LMM) analyses were performed to evaluate longitudinal changes in QoL, functioning, and symptom scores between baseline and follow‐up while accounting for repeated measures within individuals. Time was included as a fixed effect, with baseline treated as the reference category [53, 54].

Separate multivariable linear mixed models were constructed to identify sociodemographic and clinical factors associated with GQL, overall functioning, and general cancer‐related symptoms. Regression coefficients (β) with 95% confidence interval (CI) were reported. Statistical significance was considered at *p* < 0.05.

### 2.7 Ethics Statement

This pilot study was approved by the Institutional Review Board (IRB) and Research Ethics Committee [Ref. No.: FAHS‐REC/DIU/2021/1008(2)], Faculty of Health and Life Sciences, Daffodil International University, Dhaka, Bangladesh. All procedures were conducted in compliance with the ethical standards of the responsible committees on human experimentation (institutional and national) and with the Helsinki Declaration of 1964 and later versions. Institutional data collection permissions were also obtained from the participating hospitals. Written informed consent was obtained from all individual participants included in the study. The consent form was provided in Bengali script to ensure that participants and their caregivers could clearly comprehend the purpose of the study. The Strengthening the Reporting of Observational Studies in Epidemiology (STROBE) guidelines was followed in this study [55].

## 3 Results

### 3.1 Baseline Socio‐Demographic and Clinical Characteristics

A total of 102 patients with HNC were enrolled at baseline, with a mean age of 55.5 ± 11.4 years, and most were aged ≥ 50 years (71.6%). The cohort was predominantly male (70.6%), married (91.2%), rural residents (84.3%), and from low socioeconomic backgrounds (52.9%). Tobacco users were highly prevalent (93.1%), and nearly one‐third (29.3%) had low BMI (< 18.5 kg/m^2^).

Clinically, laryngeal cancer was the most common tumor site (29.4%), followed by pharyngeal (18.6%) and lip/oral cavity (16.7%) cancers, and two‐thirds (66.7%) of patients presented with advanced‐stage disease (III‐IV). Considering TNM staging, T2 tumors (59.8%) and nodal involvement (70.6%) were frequently observed (Table 1).

**Table 1:** Socio‐demographic and clinical characteristics.

Variable	Baseline enrolled cases (*n* = 102)	Follow‐up enrolled cases (*n* = 57)
	Frequency (%)	Frequency (%)
Age		
(*mean ± SD*)	55.5 ± 11.4	54.2 ± 10.7
25‐49 years	29 (28.4)	18 (31.6)
≥ 50 years	73 (71.6)	39 (68.4)
Gender		
Male	72 (70.6)	39 (68.4)
Female	30 (29.4)	18 (31.6)
Marital status		
Married	93 (91.2)	51 (89.5)
Single and others	9 (8.8)	6 (10.5)
Educational status		
Illiterate	33 (32.4)	19 (33.3)
Up to primary	44 (43.1)	24 (42.1)
≥ High school	25 (24.5)	14 (24.6)
Employment status		
Employed	68 (66.7)	32 (61.4)
Unemployed	34 (33.3)	22 (38.6)
Residential location		
Rural	86 (84.3)	48 (84.2)
Urban	16 (15.7)	9 (15.8)
Socioeconomic status		
Lower	54 (52.9)	27 (47.4)
Lower middle and above	48 (47.1)	30 (52.6)
Tobacco exposure		
Yes	95 (93.1)	52 (91.2)
No	7 (6.9)	5 (8.8)
Body mass index		
(*mean ± SD*)	20.02 ± 2.66	20.21 ± 2.66
Underweight (< 18.5 kgm^−2^)	30 (29.4)	14 (24.6)
Normal (18.5–24.9 kgm^−2^)	72 (70.6)	43 (75.4)
Disease location		
Lip and oral cavity	17 (16.7)	13 (22.8)
Pharynx	19 (18.6)	11 (19.3)
Larynx	30 (29.4)	15 (26.3)
Others	36 (35.3)	18 (31.6)
T stage		
T1	10 (9.8)	6 (10.5)
T2	61 (59.8)	39 (68.4)
T3	25 (24.5)	7 (12.3)
T4	6 (5.9)	5 (8.8)
Nodal stage		
N0	30 (29.4)	21 (36.8)
N1	55 (53.9)	29 (50.9)
N2	17 (16.7)	7 (12.3)
Metastasis stage		
M0	84 (82.4)	49 (86.0)
M1	18 (17.6)	8 (14.0)
Overall disease stage		
Stage I–II	34 (33.3)	25 (43.8)
Stage III–IV	68 (66.7)	32 (56.2)

Abbreviation: SD, standard deviation.

### 3.2 Follow‐up Outcomes and Treatment Characteristics

Follow‐up responses were obtained from 89.2% of enrolled participants. Within approximately 3 months after diagnosis, 16.5% of patients died, and 27.5% did not start CDT, including 25% of survivors. The mean duration between diagnosis and treatment initiation was 3.6 ± 1.9 weeks. Of follow‐up reached cases (*n* = 91), 57 completed the QoL assessment following a mean of 7.4 ± 2.2 weeks of treatment initiation, and the rest 45 (44.1%) were considered dropped out. Chemotherapy alone was the most common treatment regimen (40.4%), followed by mixed treatment modalities (31.6%) and surgery alone (28.1%) (Table 2).

**Table 2:** Follow‐up outcomes.

Variable	Frequency (%)
Follow‐up response by patients or their caregivers (*n* = *102*)	
Responded	91 (89.2)
Not responded	11 (10.8)
Survival outcome (*n* = *91*)	
Survived	76 (83.5)
Died	15 (16.5)
Treatment status of survived cases (*n* = *76*)	
Received treatment	57 (75.0)
Not received treatment	19 (25.0)
Treatment status of death cases (*n* = *15*)	
Received treatment	9 (60)
Not received treatment	6 (40)
Overall treatment received status (*n* = 91)	
Received treatment	66 (72.5)
Not received treatment	25 (27.5)
Treatment modalities received by follow‐up enrolled participants (*n* = *57*)	
Surgical only	16 (28.1)
Chemotherapy only	23 (40.4)
Mixed intervention	18 (31.6)
Time between diagnosis and treatment initiation (weeks)	
(*mean ± SD*)	3.6 ± 1.9 (Range: 1–8)
Time between treatment initiation and follow‐up (weeks)	
(*mean ± SD*)	7.4 ± 2.2 (Range: 2–12)

Abbreviation: SD, Standard deviation.

### 3.3 Changes in QoL, Functioning, and Symptoms

Linear mixed‐model analysis showed no significant change in deteriorated GQL scores over time (β = −0.15, *p* = 0.95). However, overall functioning deteriorated significantly, primarily mediated by declines in physical (β = −12.98, *p* = 0.001) and role (β = −11.70, *p* = 0.001) functioning following CDT initiation (β = −5.87, *p* = 0.03). Changes in emotional, cognitive, and social functioning were not statistically significant.

The symptom burden substantially increased at early assessment after CDT initiation. Significant worsening observed for fatigue (β = 9.94, *p* = 0.004), nausea/vomiting (β = 14.04, *p* = 0.003), and insomnia (β = 11.70, *p* = 0.04). Conversely, pain significantly improved over time (β = −11.11, *p* = 0.009). The burden of overall general cancer‐related symptoms (GC‐RS) increased over time, with borderline significance (β = 4.29, *p* = 0.05), indicating a continued worsening symptom profile (Table 3).

**Table 3:** Early changes in EORTC QLQ‐C30 domains (QoL, functioning, symptom burden) using linear mixed‐model analysis (*n* = 57).

Covariate	Time	Median score (Q1, Q3)	Mean score (SD)	Regression coefficient (β) between follow‐up and baseline (Ref.) (95% C.I.)	*p*‐value* (follow‐up *vs.* baseline)
Global quality of life†	Baseline	33.33 (20.83, 50.00)	33.33 (15.51)	Ref.	
	Follow‐up	33.33 (16.67, 45.83)	33.19 (20.26)	−0.15 (−4.68, 4.38)	0.95
Overall functioning†	Baseline	67.00 (53.17, 76.33)	62.63 (18.89)	Ref.	
	Follow‐up	57.33 (43.00, 74.00)	56.75 (21.26)	−5.87 (−11.17, −0.57)	0.03
Overall GC‐RS††	Baseline	29.01 (19.44, 44.44)	32.17 (14.28)	Ref.	
	Follow‐up	37.04 (21.30, 50.62)	36.46 (17.04)	4.29 (−0.14, 8.72)	0.05
Functioning scales†					
Physical functioning	Baseline	86.67 (53.33, 100.00)	72.63 (29.27)	Ref.	
	Follow‐up	60.00 (46.67, 86.67)	59.65 (29.45)	−12.98 (−20.37, −5.59)	0.001
Role functioning	Baseline	66.67 (33.33, 83.33)	57.02 (26.53)	Ref.	
	Follow‐up	50.00 (33.33, 66.67)	45.32 (27.23)	−11.70 (−18.11, −5.28)	0.001
Emotional functioning	Baseline	50.00 (33.33, 66.67)	48.98 (19.61)	Ref.	
	Follow‐up	50.00 (37.50, 75.00)	52.78 (24.72)	3.80 (−3.62, 11.22)	0.31
Cognitive functioning	Baseline	83.33 (75.00, 100.00)	84.50 (19.12)	Ref.	
	Follow‐up	83.33 (66.67, 100.00)	79.24 (18.97)	−5.26 (−11.18, 0.66)	0.08
Social functioning	Baseline	33.33 (50.00, 66.67)	50.00 (24.19)	Ref.	
	Follow‐up	33.33 (33.33, 66.67)	46.78 (24.89)	−3.22 (−9.61, 3.17)	0.32
Symptom scales††					
Fatigue	Baseline	44.44 (22.22, 61.11)	44.24 (26.18)	Ref.	
	Follow‐up	55.56 (33.33, 66.67)	54.19 (27.38)	9.94 (3.36, 16.53)	0.004
Nausea and vomiting	Baseline	0.00 (0.00, 0.00)	6.14 (14.98)	Ref.	
	Follow‐up	0.00 (0.00, 33.33)	20.18 (30.82)	14.04 (6.07, 21.99)	0.003
Pain	Baseline	50.00 (50.00, 66.67)	56.73 (21.10)	Ref.	
	Follow‐up	50.00 (25.00, 66.67)	45.61 (29.13)	−11.11 (−19.31, −2.91)	0.009
Dyspnea	Baseline	0.00 (0.00, 0.00)	9.36 (19.67)	Ref.	
	Follow‐up	0.00 (0.00, 0.00)	10.53 (23.70)	1.17 (−3.55, 5.89)	0.62
Loss of appetite	Baseline	0.00 (0.00, 33.33)	22.81 (32.83)	Ref.	
	Follow‐up	33.33 (0.00, 66.67)	30.41 (31.67)	7.60 (−3.23, 18.44)	0.17
Constipation	Baseline	66.67 (0.00, 66.67)	40.35 (34.35)	Ref.	
	Follow‐up	33.33 (0.00, 66.67)	38.60 (34.95)	1.75 (−11.84, 8.33)	0.73
Diarrhea	Baseline	0.00 (0.00, 0.00)	0.00 (0.00)		
	Follow‐up	0.00 (0.00, 0.00)	8.19 (22.08)		
Insomnia	Baseline	66.67 (0.00, 66.67)	43.27 (38.81)	Ref.	
	Follow‐up	66.67 (33.33, 83.33)	54.97 (34.21)	11.70 (0.06, 23.34)	0.04
Financial difficulties	Baseline	66.67 (0.00, 66.67)	66.67 (30.21)	Ref.	
	Follow‐up	66.67 (66.67, 100.00)	69.01 (28.07)	2.34 (−5.83, 10.50)	0.57

*Note:* EORTC QLQ‐C30: European Organization for Research and Treatment of Cancer Quality of Life Questionnaire Core 30, GC‐RS: general cancer‐related symptom, Median score (Q1, Q3): Median score with 25th and 75th interquartile ranges), QoL: Quality of life, Ref.: Referent, SD: standard deviation. **†**Higher scores reflect better QoL and functioning, **††**Higher scores reflect more symptom burden, *Significant at 5% level of significance.

### 3.4 Factors Associated With QoL, Functioning, and Symptom Burden

Follow‐up time was independently associated with deterioration in overall functioning after adjustment for demographic and clinical factors (β = −5.87, *p* = 0.03). Advanced‐stage disease was independently associated with higher GC‐RS burden (β = −9.02, *p* = 0.03). The linear effect of time also showed a tendency to be positively correlated with worsened GC‐RS burden (β = 4.29, *p* = 0.06). Similarly, low BMI demonstrated a tendency to be positively associated with deteriorated GQL (β = 9.63, *p* = 0.08) and declined overall functioning (β = 12.32, *p* = 0.05). No factors showed a significant association with GQL in this adjusted intermediate model (Table 4).

**Table 4:** Effects of sociodemographic and clinical factors over QoL, functioning, and general cancer‐related symptoms (*n* = 57).

Independent variable	Global quality of life	*p*‐value*	Overall functioning	*p*‐value*	GC‐RS	*p*‐value*
	Coefficient [β] (95% CI)		Coefficient [β] (95% CI)		Coefficient [β] (95% CI)	
Intercept	14.60 (−8.74, 37.95)	0.21	38.30 (10.89, 65.71)	0.007	57.55 (37.06, 78.05)	< 0.001
Age (< 50 years)	5.54 (−4.75, 15.83)	0.28	−0.42 (−12.41, 11.57)	0.94	0.29 (−8.61, 9.20)	0.95
Gender (male)	−13.39 (−37.53, 10.73)	0.27	−20.44 (−48.55, 7.67)	0.15	13.22 (−7.66, 34.10)	0.21
Married	−0.56 (−17.27, 16.15)	0.95	5.02 (−14.44, 24.48)	0.61	−6.36 (−20.82, 8.09)	0.38
Education (illiterate)	2.89 (−7.22, 12.99)	0.57	−1.47 (−13.23, 10.30)	0.80	−0.44 (−9.18, 8.31)	0.92
Employed	15.59 (−6.41, 37.58)	0.16	19.21 (−6.41, 44.84)	0.14	−14.75 (−33.78, 4.29)	0.13
Residence (rural)	−2.11 (−15.11, 10.89)	0.75	−3.61 (−18.75, 11.53)	0.63	−3.35 (−14.60, 7.89)	0.55
Socioeconomic status (lower)	−0.33 (−9.29, 8.63)	0.94	2.34 (−8.09, 12.78)	0.65	−3.65 (−11.41, 4.10)	0.35
Tobacco user	12.62 (−2.91, 28.15)	0.11	14.60 (−3.49, 32.69)	0.11	−8.08 (−21.52, 5.36)	0.23
Body mass index (< 18.5 kg/m^2^)	9.63 (−1.15, 20.41)	0.08	12.32 (−0.24, 24.88)	0.05	−7.26 (−16.59, 2.07)	0.12
Disease stage (III–IV)	7.25 (−2.09, 16.58)	0.13	8.76 (−2.12, 19.64)	0.11	−9.02 (−17.11, −0.93)	0.03
Disease location						
Lip and oral cavity (vs. others)	1.55 (−11.34, 14.43)	0.81	9.69 (−5.32, 24.69)	0.20	−1.89 (−13.05, 9.26)	0.73
Pharynx (vs. others)	−0.41 (−13.11, 12.28)	0.95	7.51 (−7.28, 22.30)	0.31	−5.34 (−16.32, 5.65)	0.33
Larynx (vs. others)	−0.17 (−12.02, 11.67)	0.98	8.00 (−5.79, 21.80)	0.25	−5.73 (−15.98, 4.52)	0.27
Treatment						
Surgery (vs. mixed)	−0.45 (−12.65, 11.75)	0.94	3.85 (−10.36, 18.07)	0.59	−5.33 (−15.89, 5.23)	0.31
Chemotherapy (vs. mixed)	0.52 (−10.09, 11.12)	0.92	5.93 (−6.42, 18.28)	0.34	−2.50 (−11.64, 6.72)	0.59
Time	−0.15 (−4.68, 4.38)	0.95	−5.87 (−11.17, −0.57)	0.03	4.29 (−0.14, 8.72)	0.06
Model summary	−2 Log Likelihood: 852.35; Akaike's Information Criterion (AIC): 860.35; Schwarz's Bayesian Criterion (BIC): 870.65		−2 Log Likelihood: 882.47; Akaike's Information Criterion (AIC): 890.47; Schwarz's Bayesian Criterion (BIC): 900.77		−2 Log Likelihood: 837.94; Akaike's Information Criterion (AIC): 845.94; Schwarz's Bayesian Criterion (BIC): 856.23	

*Note:* *Significant at 5% level of significance.

Abbreviations: C.I., confidence interval; GC‐RS, general cancer‐related symptoms; QoL, quality of life; versus, versus.

### 3.5 Independent Predictors of Global Quality of Life

In multivariable mixed‐effects model, younger than 50 years of age was a significant independent predictor of higher GQL (β = 6.43, *p* = 0.005). Patients with pharyngeal (β = −6.63, *p* = 0.02) and laryngeal (β = −7.33, *p* = 0.009) cancers showed poorer QoL compared to other disease sites.

Better social functioning was positively associated with QoL (β = 0.15, *p* = 0.02), whereas fatigue (β = −0.15, *p* = 0.04), pain (β = −0.10, *p* = 0.03), and financial difficulties (β = −0.09, *p* = 0.02) were significant negative predictors of QoL (Table 5).

**Table 5:** Effects of sociodemographic and clinical factors and symptom burdens over QoL using multivariable linear mixed‐model (*n* = 57).

Independent variable	Standard error	Degree of freedom	t‐statistic	*p*‐value*	Coefficient [β] (95% Confidence interval)
Intercept	12.64	112.25	2.60	0.01	32.84 (7.79, 57.90)
Age (< 50 years)	2.17	53.60	2.96	0.01	6.43 (2.07, 10.78)
Gender (male)	5.36	57.45	0.21	0.84	1.10 (−9.63, 11.83)
Married	3.72	58.15	−1.24	0.22	−4.61 (−12.06, 2.84)
Education (illiterate)	2.21	56.68	1.54	0.13	3.39 (−1.03, 7.81)
Employed	5.12	63.12	0.37	0.71	1.90 (−8.34, 12.14)
Residence (rural)	3.05	66.85	0.19	0.85	0.59 (−5.49, 6.67)
Socioeconomic status (lower)	2.09	61.36	−1.74	0.09	−3.64 (−7.81, 0.54)
Tobacco user	3.71	64.80	0.98	0.33	3.63 (−3.78, 11.03)
Body mass index (< 18.5 kg/m^2^)	2.43	58.97	0.90	0.37	2.20 (−2.66, 7.06)
Disease stage (III–IV)	2.05	57.50	−0.15	0.88	−0.31 (−4.42, 3.79)
Disease location					
Lip and oral cavity (vs. others)	3.11	62.64	−1.27	0.21	−3.94 (−10.15, 2.28)
Pharynx (vs. others)	2.85	57.80	−2.33	0.02	−6.63 (−12.33, −0.92)
Larynx (vs. others)	2.71	59.77	−2.70	0.009	−7.33 (−12.75, −1.90)
Treatment					
Surgery (vs. mixed)	2.81	62.99	−0.41	0.68	−1.16 (−6.77, 4.45)
Chemotherapy (vs. mixed)	2.45	62.78	−1.40	0.17	−3.42 (−8.30, 1.47)
Physical functioning	0.06	97.99	1.22	0.22	0.07 (−0.05, 0.19)
Role functioning	0.06	106.16	0.22	0.83	0.01 (−0.11, 0.13)
Emotional functioning	0.06	102.10	0.22	0.83	0.01 (−0.11, 0.14)
Cognitive functioning	0.07	104.88	1.37	0.17	0.09 (−0.04, 0.22)
Social functioning	0.06	89.33	2.45	0.02	0.15 (0.03, 0.26)
Fatigue	0.07	101.92	−2.07	0.04	−0.15 (−0.29, −0.01)
Nausea and vomiting	0.05	74.32	1.04	0.30	0.05 (−0.04, 0.14)
Pain	0.05	113.68	−2.15	0.03	−0.10 (−0.19, −0.01)
Dyspnea	0.05	106.87	−1.06	0.29	−0.05 (−0.15, 0.05)
Loss of appetite	0.03	100.78	−0.25	0.80	−0.01 (−0.07, 0.05)
Constipation	0.03	103.82	−1.65	0.10	−0.05 (−0.11, 0.01)
Diarrhea	0.07	60.93	0.08	0.94	0.01 (−0.14, 0.15)
Insomnia	0.03	104.82	−0.28	0.78	−0.01 (−0.07, 0.05)
Financial difficulties	0.04	86.36	−2.33	0.02	−0.09 (−0.17, −0.01)
Time	1.93	79.55	0.98	0.33	1.88 (−1.95, 5.72)
Model summary	−2 Log Likelihood: 804.24; Akaike's Information Criterion (AIC): 874.24; Schwarz's Bayesian Criterion (BIC): 970.01				

*Note:* *Significant at 5% level of significance.

Abbreviation: vs, versus.

## 4 Discussion

To our knowledge, this is the first kind of study in Bangladesh to assess early changes in QoL, functioning, and symptom burden using the EORTC QLQ‐C30 among patients with HNC following CDT initiation. The study demonstrated substantial deterioration in functioning and increased symptom severity within 7 weeks (mean duration) after initiation of CDT, despite moderate improvement in pain scores. These findings indicate a considerable short‐term treatment burden among Bangladeshi patients with HNC.

A dropout rate of 44% was observed in the cohort within a short‐term follow‐up after CDT initiation, exceeding the 25%–42% nonparticipation reported in relevant studies at 1‐year follow‐up [56, 57]. Additionally, observed early mortality rates surpassed those shown in related studies, which ranged from 20% to 23.2% within 2‐year follow‐up periods [17, 19, 37, 43]. All these reflect severe disease burden, treatment delays, deteriorating physical condition, and socioeconomic barriers affecting treatment continuation and follow‐up [47, 49, 58, 59, 60].

The cohort was predominantly male, tobacco‐exposed, and diagnosed at advanced stages, consistent with previous studies in Asian countries [2, 3, 4, 5, 6, 18, 26, 27, 34, 35, 36, 37, 38, 39, 42]. The high prevalence of advanced‐stage disease among newly confirmed HNC cases may reflect prolonged disease identification, weak awareness, limited access to specialized services, financial adversities or poverty, belief in traditional healing practices, and an infantile early cancer screening system in Bangladesh [4, 5, 8, 9, 11, 12, 29, 37, 38, 49, 59, 60].

The current findings revealed a significant deterioration in overall functioning, primarily due to a decline in physical and role functioning, at an early follow‐up after CDT initiation. Similar declines in functioning during early treatment phases have been reported in previous longitudinal studies among patients with HNC [16, 19, 20, 25, 29, 40, 42]. Longitudinal studies showed that functional domains tend to worsen early after CDT initiation and sometimes take 1‐2 years to return to pretreatment status [19, 25, 40]. Patients receiving multimodal CDT experience greater functional deterioration, tailored disease sites and severity, and cumulative toxicities associated with surgery, chemotherapy and radiation therapy [16, 18, 19, 20, 25, 40, 42]. Patients frequently experience impaired speech, chewing, swallowing, and nutritional intake, thereby negatively affecting daily functioning and QoL [18, 21, 26, 27, 42].

Symptom burden impacts greater HR‐QoL changes within the first 3–4 months of treatment [34, 61]. The current findings showed a significantly increased symptom burden at an early follow‐up, with fatigue, nausea/vomiting, and insomnia indicating worsening QoL. Fatigue emerged as an independent negative predictor of QoL, which is consistent with other studies that identified severe fatigue as one of the determinants of reduced HR‐QoL among patients with HNC [18, 23, 40, 42, 61]. However, significant improvement in pain symptoms at an early follow‐up after CDT initiation in our study may reflect treatment response. Financial difficulties also found to be an independent predictor of poorer QoL, highlighting the major economic burden of cancer care in low‐resource settings like Bangladesh.

Evidence from Bangladesh showed that the poorer health outcomes have been linked to symptom burden, financial hardship, advanced‐stage cancer, physical inactivity, and severe malnutrition among mixed‐cancer patients [48, 49, 50, 51]. Similarly, current findings indicate that advanced‐stage disease correlates with higher symptom burden, whereas lower BMI shows a tendency toward poorer QoL and functioning. These findings emphasize the clinical importance of nutritional status, early‐stage diagnosis, and supportive care in improving patient outcomes. Additionally, social functioning positively influenced GQL, underscoring the role of family and social support during treatment.

This study has several limitations. The relatively small sample size and substantial loss to follow‐up may have influenced statistical power and limited treatment‐specific comparisons. As the longitudinal QoL analyses were restricted to participants who survived and completed follow‐up, the exclusion of patients who died or were lost to follow‐up may have resulted in some underestimation of poorer QoL outcomes and symptom burden in the overall cohort. Although the study's pilot and exploratory nature allowed for an early assessment, it limited the capture of long‐term effects on QoL. Additionally, detailed information regarding chemotherapy regimens and radiation doses could not be collected. Participants who completed the follow‐up assessment were still undergoing treatment and had not completed their planned therapy courses, further, limiting meaningful treatment‐specific comparisons. However, the multicenter design and longitudinal assessment provide important preliminary evidence regarding QoL trajectories among patients with HNC in Bangladesh.

## 5 Conclusion

Patients with HNC in Bangladesh experienced marked deterioration in functioning and increased symptom burden during the early post‐treatment period, despite modest improvement in pain. Fatigue, financial difficulties, advanced‐stage disease, and poorer social functioning were important determinants of deteriorated QoL. Additionally, higher mortality and treatment gaps are marked at an early follow‐up. These findings emphasize the necessity of integrated supportive care strategies, including symptom management, social counselling, and financial assistance during cancer treatment, alongside efforts to address treatment gaps. Larger longitudinal studies with long follow‐ups are warranted to better understand long‐term QoL trajectories and to inform policy implications and implementation regarding early identification and treatment of HNC in Bangladesh.

## Author Contributions

**Md. Jahirul Islam:** conceptualization, methodology, data curation, investigation, validation, formal analysis, supervision, writing – original draft, writing – review and editing. **A.B.M. Alauddin Chowdhury:** conceptualization, methodology, data curation, investigation, validation, supervision, writing – review and editing. **Md. Imdadul Haque:** conceptualization, methodology, data curation, investigation, validation, formal analysis, supervision, writing – review and editing. **Md. Khalid Mahmud:** conceptualization, methodology, data curation, investigation, validation, supervision, writing – review and editing. **Kawsar Ahmed:** conceptualization, methodology, data curation, investigation, validation, formal analysis, writing – review and editing. **Hafiz T.A. Khan:** conceptualization, methodology, writing – review and editing, validation, supervision. **Salim Khan:** conceptualization, methodology, data curation, investigation, validation, supervision, writing – review and editing.

## Funding

The authors have nothing to report.

## Ethics Statement

This study was performed in line with the principles of the Declaration of Helsinki. Approval was granted by the Research Ethics Committee [Ref. No.: FAHS‐REC/DIU/2021/1008(2)], Faculty of Health and Life Sciences, Daffodil International University, Dhaka, Bangladesh.

## Consent to Participate

Written informed consent was obtained from all individual participants included in the study.

## Consent to Publish

All participants also provided informed consent regarding the publication of their data.

## Conflicts of Interest

The authors declare no conflicts of interest.

## Transparency Statement

All authors have read and approved the final version of the manuscript. All authors had full access to all of the data in this study, and Md. Jahirul Islam [corresponding author] takes complete responsibility for the integrity and accuracy of the data. The corresponding author also affirms that this manuscript is an honest, accurate, and transparent account of the study being reported; that no important aspects of the study have been omitted; and that any discrepancies from the study as planned have been explained.

## Data Availability

The data that support the findings of this study are openly available in Mendeley Data at https://data.mendeley.com/datasets/r9r9k96f5x/1. The datasets generated and/or analyzed during the current study are available in the Mendeley Data repository, web link: https://data.mendeley.com/datasets/r9r9k96f5x/1.
